# “Stocking Pattern Metabolic Captivity” of Legs on ^18^ F-FDG PET-CT in Necrotizing Fasciitis: Potential Complimentary Role in Differential Diagnosis and Assessment of Disease Extent in a Life-Threatening Condition

**DOI:** 10.1055/s-0042-1757289

**Published:** 2022-10-28

**Authors:** Sunita Nitin Sonavane, Sandip Basu

**Affiliations:** 1 Radiation Medicine Centre, Bhabha Atomic Research Centre, Tata Memorial Hospital, Mumbai, Maharashtra, India; 2 Homi Bhabha National Institute, Mumbai, Maharashtra, India

**Keywords:** FDG, PET-CT, necrotizing fasciitis, paraneoplastic syndrome

## Abstract

A rare and fatal life-threatening case of necrotizing fasciitis (initially presenting with skin-deep superficial lesions and clinical suspicion of paraneoplastic syndrome) is described, who was finally diagnosed with the help of fluorodeoxyglucose-positron emission tomography (FDG-PET)/computed tomography (CT) as more extensive infectious process. A 36-year-old male presented with bilaterally symmetrical cutaneous lesions involving lower limbs that rapidly progressed to ulcerative lesions and pancytopenia. In view of suspicion of paraneoplastic manifestation, the patient underwent ^18^ F-FDG-PET/CT to rule out any underlying malignancy. The FDG-PET/CT findings confirmed hypermetabolism circumferentially along the fasciae of bilateral lower extremities while sparing muscles and subcutaneous fat from below the knee till toe with diffused hypermetabolic marrow, and no evidence of focal disease suggesting malignancy. Biopsy turned out to be superficial necrolytic fasciitis. The patient's condition deteriorated and, 20 days following the scan, the patient succumbed secondary to severe pancytopenia and hypotension. The case raises the importance of high degree of suspicion and prompt diagnosis of this condition, where FDG-PET/CT imaging can play a valuable complimentary role. Such awareness could be lifesaving due to early optimal treatment in the disease course.

## Introduction

Necrotizing fasciitis (NF) is a rapidly progressing and potentially life-threatening infectious process of the fascia, perifascial planes, and can also cause a secondary involvement of the overlying and underlying skin, soft tissue, and muscle. 1 2 Polymicrobial infections can occur because of a combination of gram-negative and anaerobic involvement. 3 NF affects about 0.4 in every 100,000 people per year in the United States, while in some parts of the world, it is as common as one in every 100,000 people. 4 The present report describes the clinical presentation, molecular fluorodeoxyglucose-positron emission tomography (FDG-PET)/computed tomography (CT) imaging, and microbiological characteristics of this condition and discusses the determinants of mortality associated with this uncommon surgical emergency. 5

## Case Report

**Fig. 1:**
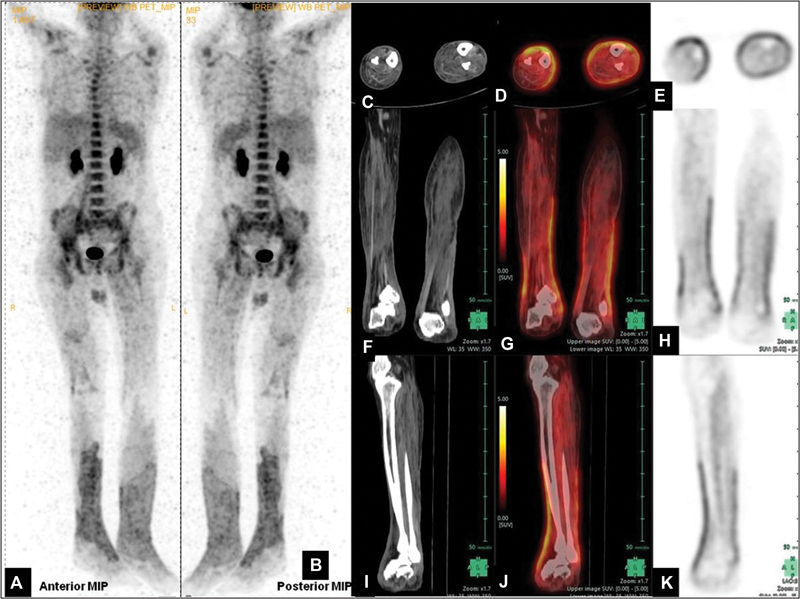
( **A** , **B** ) Maximum intensity projection (MIP) positron emission tomography (PET) image—anterior and posterior; Computed tomography (CT), PET, and fused PET/CT axial ( **C** – **E** ), coronal ( **F** – **H** ), and sagittal ( **I** – **K** ) images showing diffusely increased metabolic captivity along the fasciae sparing muscles and subcutaneous fat from the region of proximal leg to dorsum of feet bilaterally (standardized uptake value [SUV]max: 4.75). MIP ( **A** , **B** ) images reveal linear hypermetabolism in bilateral psoas muscles (right > left) (SUVmax: 6.15). Low-grade hypermetabolism accompanied by fat stranding was noted around the site of intramedullary nail insertion in diaphysis of right femur (SUVmax: 3.96). Diffuse hypermetabolic bone marrow was noted in the axial skeleton (SUVmax: 5.55). Rest of the whole-body survey was unremarkable and showed physiological tracer distribution.

**Fig. 2:**
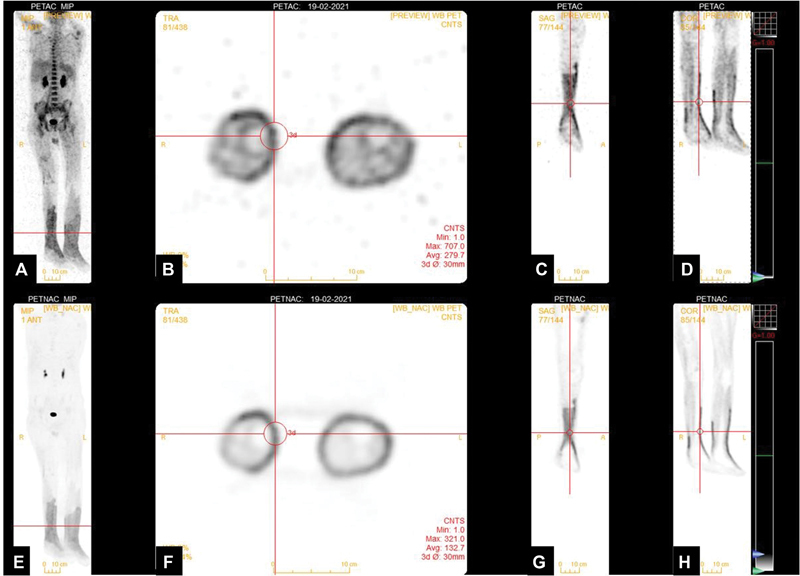
( **A** , **E** ) Anterior maximum intensity projection (MIP) images. ( **A** – **D** ) Attenuation corrected (AC) fluorodeoxyglucose-positron emission tomography (FDG-PET) images; ( **E** – **H** ) nonattenuation corrected (NAC) FDG-PET images (MIP, transaxial, coronal, and sagittal slices, respectively) reveal diffusely increased metabolic captivity along the fasciae from the region of proximal leg to dorsum of feet bilaterally.

A 36-year-old nondiabetic wheelchair-bound male, with previous history of weight loss and herpes simplex virus 2 infection, presented with bilaterally symmetrical predominantly cutaneous lesions involving extensor aspects of the lower limbs which began as multiple desquamating plaques that rapidly progressed to vesicles, bullae, and ulcers, and was suspected of pyoderma gangrenosum with peripheral neuropathy and pancytopenia. The biochemical and blood work revealed normal blood glucose but presence of anemia with hemoglobin of 6.7 g/dL, platelets of 0.9 lac/mm ^3^ , normal serum ferritin levels 84.4 (normal 20–250 ng/mL), high aspartate aminotransferase of 185 (5–40 units per liter), and preserved renal function with serum creatinine of 0.6 mg/dL (normal 0.7–1.2 mg/dL). The patient was referred for a whole body ^18^ F-FDG-PET/CT to rule out underlying malignancy considering the skin lesions as suspicious paraneoplastic syndrome. A whole-body ^18^ F-FDG-PET/CT ( Figs. 1 and 2 ) was undertaken 60 minutes after intravenous injection of 6.5 mCi of ^18^ F-FDG, using a whole-body full-ring dedicated three-dimensional PET-CT scanner (Gemini TF; 250 mAs, 120 kVp, noncontrast CT scan with 2 mm slice thickness). ^18^ F-FDG-PET/CT revealed circumferential hypermetabolism along the fasciae of bilateral lower extremities while sparing muscles and subcutaneous fat extending from below knee level till dorsum of feet (standardized uptake value [SUV]max 4.75). Linear increased FDG uptake was noted in psoas muscles bilaterally (right > left) (SUVmax 6.15) ( Figs. 1 and 2 ). Hypermetabolism accompanied by fat stranding was noted around the site of intramedullary nail insertion in diaphysis of right femur (SUVmax 3.96). Diffuse hypermetabolic bone marrow was noted (SUVmax 5.55). Non-FDG avid minimal right-sided pleural effusion with pleural thickening and adjacent infective consolidatory changes showing mild FDG uptake (SUVmax 3.19). Rest of the whole-body survey was unremarkable and showed physiological tracer distribution. Thus, there was no definite scan evidence of any focal lesion suspicious of malignancy. A diffuse increased metabolic captivity was seen from the region of proximal leg to dorsum of feet bilaterally, suggestive of active infective process; skin biopsy was performed which revealed dermal infiltrating aggregates in skin, fascia, and subcutaneous tissue mixed with lymphocytes and abundant neutrophils, small vessel vasculitis, extensive dermal glands, and fat necrosis, favoring diagnosis of superficial NF. The patient in subsequent days developed progressive extensive skin involvement extending proximally and succumbed secondary to severe pancytopenia and hypotension 20 days thereafter, raising the importance of prompt diagnosis and treatment (i.e., surgery, antibiotics, and hyperbaric chamber) of this life-threatening infection.

## Discussion

Marszał and Bielecki reported NF as mixed infection of skin and subcutaneous tissue with a characteristic clinical and pathological appearance, caused by aerobic, anaerobic, and mixed bacterial flora or may be polymicrobial with wide variety of infections, making it an increasing problem in medical and surgical practice. 6 Kihiczak et al termed NF as life-threatening condition, consisting of a soft-tissue infection that is rapidly progressive, with widespread fascial necrosis. 7 Wong et al highlighted the paucity of cutaneous findings early in the course of the disease makes the diagnosis at times difficult. 5 These investigators studied 89 patients with NF, among which diagnosis was available in 13 patients, the most common cause being streptococcus, and the most common associated comorbidity was diabetes mellitus (70.8%), advanced age, and two or more associated comorbidities. Multivariate analysis showed that only a delay in surgery of more than 24 hours was correlated with increased mortality ( *p* < 0.05; relative risk = 9.4). 5

Case reports in the literature have illustrated necrotizing dermatitis as paraneoplastic syndrome and association with multiple malignancies like chronic lymphocytic leukemia, 8 Hodgkin's lymphoma, 9 myelodysplastic syndrome, 10 metastatic endometrial cancer, 11 rectal cancer, 12 colon cancer, 13 breast carcinoma, 14 and glucagonoma. 15 In the present case, however, we were unable to delineate any malignant focus on FDG-PET/CT.

On ruling out malignancy, other conditions like immunosuppression and aplastic anemia-related association of NF is also known. Ugarte-Torres et al reported the difficult-to-treat organisms having significant implications in both clinical and public health settings. A similar case as our patient, a 37-year-old Caucasian male with immunosuppression due to aplastic anemia being treated with cyclosporine, presented to the hospital with relapsed disease, he subsequently developed overwhelming sepsis secondary to bilateral lower extremity NF. 16 The NF was caused by a multidrug-resistant strain of *Aeromonas hydrophila* . Despite broad-spectrum antibiotics and aggressive surgical debridement, he succumbed to this severe invasive infection. Kim et al in their reported case of eosinophilic fasciitis in a 37-year-old woman with a 3-month history of progressive stiffness involving her forearms and lower legs, highlighted the role of FDG-PET/CT images, FDG uptake was increased along the fasciae of bilateral upper and lower extremities while sparing muscles and subcutaneous fat, similar pattern as in the case presented in this report. Biopsy and histological examination confirmed diagnosis of eosinophilic fasciitis. The report stated FDG-PET/CT may be helpful in the diagnosis of eosinophilic fasciitis as it could clearly illustrate anatomical involvement of the disease. 17 Another PET tracer like ^68^ Ga-DOTATATE PET/CT can have diagnostic role delineating suspected glucagonoma 18 or active inflammatory processes where macrophages and leukocyte do express SSTR2 receptors. 19

With regard to considering the optimal management, Khalid et al reported necrotizing soft tissue infections being associated with considerable morbidity and mortality, and delayed recognition and treatment can have severe implications. 20 As stated previously, Kihiczak et al have reported that prompt diagnosis and treatment are essential, with surgical debridement and antibiotic therapy are the primary treatment options. 7 Marszał and Bielecki stated early and radical surgical excision of all affected tissue is the treatment of choice. 6 Adjuvant hyperbaric oxygen appeared to be important in refractory progressive bacterial gangrene. A combination of hyperbaric oxygen, surgical treatment, and antibiotics gives the lowest mortality and morbidity in gas gangrene compared with other treatment modifications. 6

## Conclusion

In summary, NF is a rapidly progressive and potentially lethal infectious condition, and delay in diagnosis can be life-threatening and a high index of suspicion and prompt management with aggressive early surgical debridement along with antibiotics as early as 24 hours of diagnosis are the most important treatment options, which can reduce mortality among these patients. The present case illustrates a young patient's irrecoverable, debilitating condition secondary to NF with illustration of a characteristic FDG-PET/CT finding in this condition.
